# Multi-modal brain data exploration software for imaging transcriptomics: A comparative evaluation and future directions

**DOI:** 10.1162/IMAG.a.1104

**Published:** 2026-01-27

**Authors:** Tobias Peherstorfer, Bianca Burger, Sophia Ulonska, Wulf Haubensak, Katja Bühler

**Affiliations:** Biomedical Image Informatics, VRVis GmbH, Vienna, Austria; Research Institute of Molecular Pathology (IMP), Vienna Biocenter (VBC), Vienna, Austria; Division of Neuronal Cell Biology, Center for Brain Research, Medical University Vienna, Vienna, Austria

**Keywords:** imaging transcriptomics, data exploration, multi-modal brain data, data integration, neuroimaging, brain mapping, hypothesis generation, data-driven neuroscience, transcriptome

## Abstract

The integration of neuroimaging and transcriptomics data has revolutionized our understanding of complex brain phenomena and holds promise for linking molecular level genetic markers to brain dynamics and function. However, the involved data differ strongly across biological scales and acquisition modalities, presenting significant preprocessing and analytical challenges. In this context, multi-modal brain data exploration software offers a low-threshold, holistic, and non-biased approach to hypothesis generation that is complementary to literature search. This review assesses current data exploration tools that combine transcriptomic and neuroimaging data, evaluating them based on species coverage, data modalities, data integration approaches, visualization capabilities, and user data integration. We highlight the potential of these tools by discussing their applicability for example research questions and identify yet unaddressed workflows and promising approaches to support imaging transcriptomics analyses. This comprehensive survey aims to inform researchers in their selection of suitable software for their specific research needs and to guide the development of more effective multi-modal brain data exploration tools.

## 1 Introduction

Linking genes to neuronal networks and, ultimately, brain function is a central goal from basic neuroscience research to comparative neuroanatomy and translational psychiatry. In recent years, the number of publically available neuroscientific data resources has grown dramatically, which not only allows for discoveries supported by large sample sizes but also for multi-modal data integration approaches (Amunts et al., 2024). Indeed, several studies have concluded that complex neuroscientific phenomena are best studied using multi-modal data fusion (Calhoun & Sui, 2016; Timonidis et al., 2021; Walter et al., 2020). This is demonstrated by the success of multi-modal neuroscience, where new insights are achieved by linking modalities such as (electro)physiology, transcriptomics, connectivity, and morphology (Arkhipov et al., 2025). A growing number of publications has presented novel insights by investigating relationships between spatial gene expression patterns and variations in the brain’s structural and functional architecture (Cai et al., 2024; Forest et al., 2017; Fornito et al., 2019; Fulcher & Fornito, 2016; Goel et al., 2014; Ji et al., 2014; Patania et al., 2019; Richiardi et al., 2015; Romero-Garcia et al., 2018; Xia et al., 2022), as well as neurodevelopment (Dear et al., 2024). This specific type of relational analysis has coined the name of the burgeoning field of imaging transcriptomics (Mandal et al., 2022; Martins et al., 2021), which is concerned with the identification of spatial correlations between gene expression patterns and properties of brain structure and function. As such, it is a subfield of multi-modal neuroscience and provides powerful tools for linking image-derived phenotypes to genomic mechanisms (Ecker et al., 2025). For an overview of imaging transcriptomics of brain disorders, we refer to Arnatkeviciute et al. (2022).

In general, imaging transcriptomics approaches start with a hypothesis, for example, a suspected relationship between regional gray matter volume and sex chromosome gene expression (S. Liu et al., 2020). These hypotheses are often generated through literature review, but can also originate directly from inspecting trends and patterns in data. While the integration of multi-modal data to test such hypotheses can further our understanding of the brain, it also opens up new challenges (Arnatkeviciute et al., 2023). Imaging transcriptomics analyses require extensive pre-processing of neuroimaging and transcriptomics data (Arnatkeviciute et al., 2019). Both data need to be registered to the same standard space (Oliveira & Tavares, 2014). Often, dimensionality reduction techniques or gene filtering is used to simplify transcriptomics data for downstream analysis techniques (Xiang et al., 2021). In addition, a probe selection strategy needs to be chosen and implemented for biopsy-based gene expression modalities (Arnatkeviciute et al., 2019). Finally, transcription–neuroimaging association analysis is done using clustering or correlative approaches (Cai et al., 2024; Forest et al., 2017; Goel et al., 2014; Ji et al., 2014; S. Liu et al., 2020; Richiardi et al., 2015). For a more detailed discussion of these steps, we refer to Arnatkeviciute et al. (2023). These steps are typically performed by bioinformaticians, typically employing ad hoc designed software and analysis workflows case by case. Considerable time and resources have to be invested for such analyses. Therefore, hypothesis-driven imaging transcriptomics approaches have to be based on carefully chosen, promising hypotheses. Moreover, the intricacy involved in accessing, processing, integrating, and analyzing transcriptomic data hinders the utilization and re-use of these data resources.

We argue that researchers can use existing brain data exploration and mining tools for low-threshold, holistic, and non-biased hypothesis generation using already integrated multi-modal brain data. Data exploration and mining are concerned with the unstructured, interactive inspection of data to gain a high-level overview of data variables and patterns and build hypotheses (Kotu & Deshpande, 2018). As such, it is an initial, investigative step to understand data, identify structures and discover data features. This is in contrast to dedicated data analysis methods, which typically succeed these exploratory workflows to build elaborate statistical models to model data, make predictions, and test hypotheses (Giacomel et al., 2022; Martins et al., 2021). It is important to note that data exploration cannot be used for hypothesis testing, as it does not involve the required statistical methods. In the context of imaging transcriptomics research, data exploration software provide integrated transcriptomics and neuroimaging data, meaningful query options, and interactive visualizations in a graphical user interface. With these tools, researchers can quickly generate hypotheses by scanning multiple data modalities for patterns (e.g., visible correlation between the expression patterns of genes and functional connectivity). Thereby, hypotheses can be generated directly from exploring multi-modal data, avoiding potential biases that might be present in existing literature. Furthermore, the low-threshold visual exploration of integrated data allows introducing the world of multi-modal brain data to students without the need for programming knowledge. Multiple software that allow for such investigations have already been published. However, a holistic survey of the available exploration and mining tools is, to the best of our knowledge, not available. In an effort to inform future multi-modal brain studies, we here discuss neuroimaging and transcriptomics data exploration and mining applications, common approaches, and current shortcomings in the form of a scoping review. Thus, we provide a strategy for future surveys and a manifesto for future multi-modal brain tools.

### 1.1 Study objective and scope

The objectives of this review are:

- To review brain data exploration software for combined transcriptomics and neuroimaging research.
- To evaluate brain data exploration software based on scientists’ needs and research questions.
- To inform future developments in brain data integration and exploration.

The eligibility criteria for software to be considered in this report are:

1. The software relies on a graphical user interface.
2. The software interface is available in English.
3. The software is publicly available.
4. At least one transcriptomic and one neuroimaging brain dataset can be accessed with the software.

Constraint 1–3 ensures low-threshold access to the software, while constraint 4 is required to compare neuroimaging and transcriptomics data. Our goal is to provide a comparative overview on tools that enable a larger population of researchers to investigate imaging transcriptomics data. Specifically, we focus on tools that use interactive visualizations via a graphical user interface (GUI), combining the efficient visual information processing of humans with the capability to explore multiple levels of detail through interaction. This combination of visualization with interaction is central in the field of visual analytics. Researchers use it to augment human capabilities in processes that require a human for decision making, such as hypothesis generation (Munzner, 2014). Furthermore, we also focus on GUI-based tools because they provide generally low threshold access to data exploration without requiring any programming knowledge. Such tools support not only the re-use of complex spatial and multi-modal data resources but are also promoting data democratization, ease interdisciplinary cooperation, and can be valuable assets in education.

## 2 Term Definition and Search Methodology

Before discussing existing brain data integration and exploration platforms, we first introduce common brain data integration concepts and briefly discuss our literature search.

### 2.1 Templates and reference spaces

Templates are standard brain images that other data are aligned to, making them comparable (Evans et al., 2012). They can be divided into 2D and 3D templates. Many 2D templates are built from sequential brain slice images in coronal, sagittal, or horizontal direction, but 2D flat maps representing the highly convolved brain surface are also widely used. 3D template brains are often single-subject or group-averaged volumetric images and can have symmetric or asymmetric brain hemispheres (Fonov et al., 2011; Mazziotta et al., 2001). In species with smaller brains (e.g., mice), templates are commonly reconstructed from series of 2D slice images. Popular templates include the MNI152 nonlinear for the human (Fonov et al., 2011) and the template of the Allen Reference Atlas for the mouse, available at http://atlas.brain-map.org/. The coordinate space in which templates are defined is commonly called reference space. For a thorough discussion of templates and reference spaces, we refer to Evans et al. (2012).

### 2.2 Reference atlas and parcellation

**Fig. 1.**
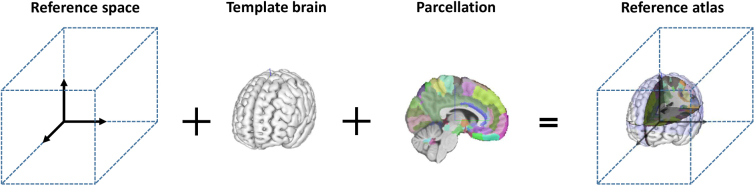
Schematic ingredients of a reference atlas. Brain renderings were taken from SIIBRA Explorer.

A brain consists of regions with distinct anatomical, functional, and other properties and can be annotated and named on a template representation of the brain. A group of labeled and annotated brain regions of the same type is called brain parcellation and is usually hierarchically organized and complete, that is, it covers the entire brain without gaps. For a comprehensive review of imaging-based parcellation of the human brain, we refer to Eickhoff et al. (2018). The combination of reference space, template, and brain parcellation is commonly referred to as a reference atlas (see Fig. 1) (Evans et al., 2012; Timonidis et al., 2021). The concept of parcellations based on cytoarchitecture has been used since the early 20th century (Brodmann, 1909). Nowadays, parcellations using, for example, functional regions (Dadi et al., 2020), white matter tracts (P. Guevara et al., 2012), cortical layers (Wagstyl et al., 2020), or cytoarchitecture (Amunts et al., 2020) are available. Commonly used reference atlases involve the Talairach atlas (Talairach et al., 1988), the Allen Human Brain Reference Atlas (Ding et al., 2016), and the Allen Mouse Brain Common Coordinate Framework (CCF) (Wang et al., 2020). A more detailed discussion of human brain atlases is given by Evans et al. (2012) and Hess et al. (2018).

### 2.3 Data integration across resolution levels

**Fig. 2.**
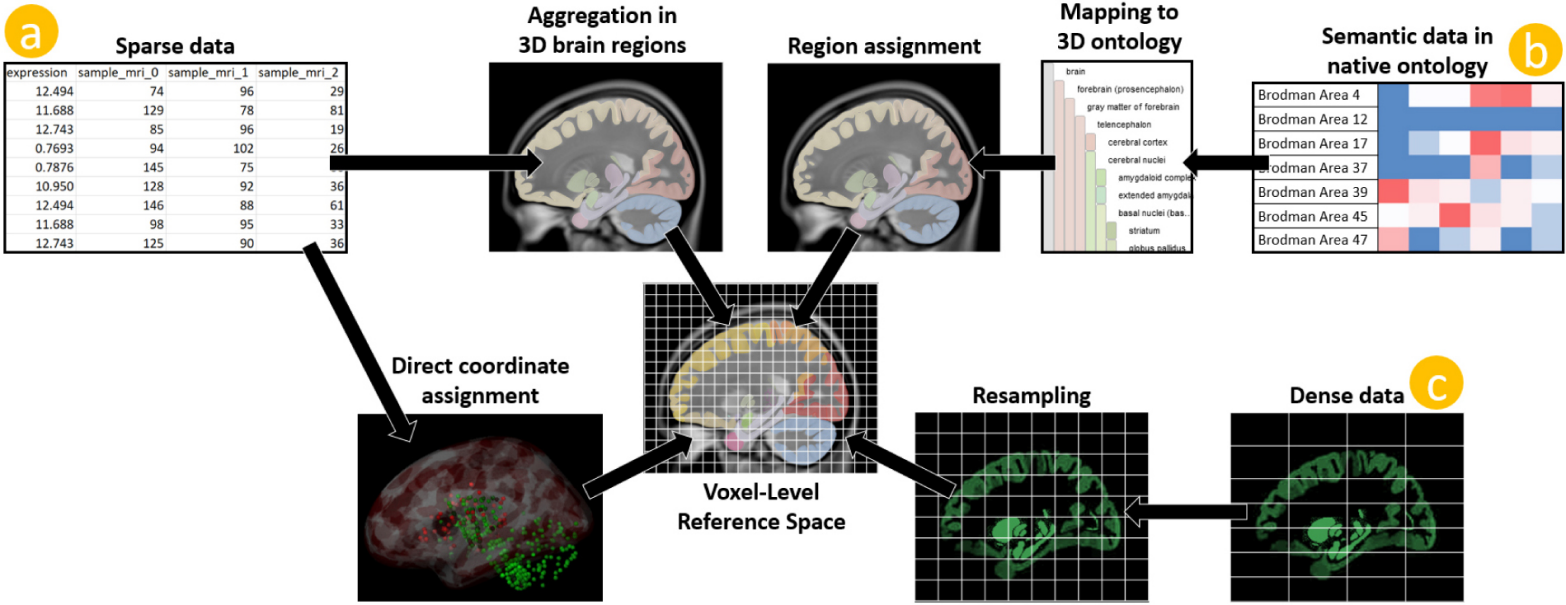
Schematic of common data integration approaches for dense, sparse, and semantic spatial brain data. Dense data (c) are resampled to fit the reference space resolution. Sparse data (a) are converted to dense data, either by aggregating it in brain regions or assigning directly to volume space. Semantic data (b) are mapped from its native brain region ontology to a 3D ontology and assigned to brain region volumes. Data visualizations were taken from BrainTrawler and the Allen Brain website.

The spatial resolution of brain data varies strongly with the underlying acquisition technique (e.g., single cell sequencing, spatial transcriptomics, electrophysiology, magnetic resonance imaging). Commonly, imaging- and microscopy-based methods, such as functional magnetic resonance imaging (fMRI) or in-situ hybridization (ISH), are processed into volumetric data, with one value for each voxel in a 3D grid. In contrast, biopsy-site based methods, such as RNA sequencing, record data for specific brain coordinates. Exact coordinates are often omitted, for example, in functional connectivity data, where data are instead labeled with brain regions for noise and complexity reduction and increased interpretability. In this publication, we chose to categorize voxel-resolution, sample-resolution, and brain region-resolution spatial brain data as *dense*, *sparse*, and *semantic* data, respectively. To integrate these data types, they need to be unified in a common reference space, as illustrated in Figure 2. Dense data are commonly brought to the same spatial resolution as the reference space by resampling and interpolation. For semantic data, the native ontology needs to be mapped to a brain parcellation with 3D volumes assigned to each parcellation region. These volumes can then be used to map semantic data to the reference space (Arnatkeviciute et al., 2019). Multiple options for the integration of sparse data exist. Apart from direct assignment of sparse data to volume space (Mesmoudi et al., 2015), a common approach is to convert it into dense data by aggregation in 3D brain regions (Arnatkeviciute et al., 2019). Direct assignment to volume space is commonly followed up by assigning the respective values of a sample to all voxels in a sphere around sample locations (A. S. Fox et al., 2014; Z. Liu et al., 2019) for downstream analysis. The choices for the integration of semantic and sparse data can severely affect data findability and can, if not communicated correctly, convey false impressions of the underlying data. In addition to spatial brain data, it is often useful to link non-spatial brain data, such as gene annotations, to the corresponding spatial data to allow interactive searching and exploration of the data.

### 2.4 Search methodology

**Fig. 3.**
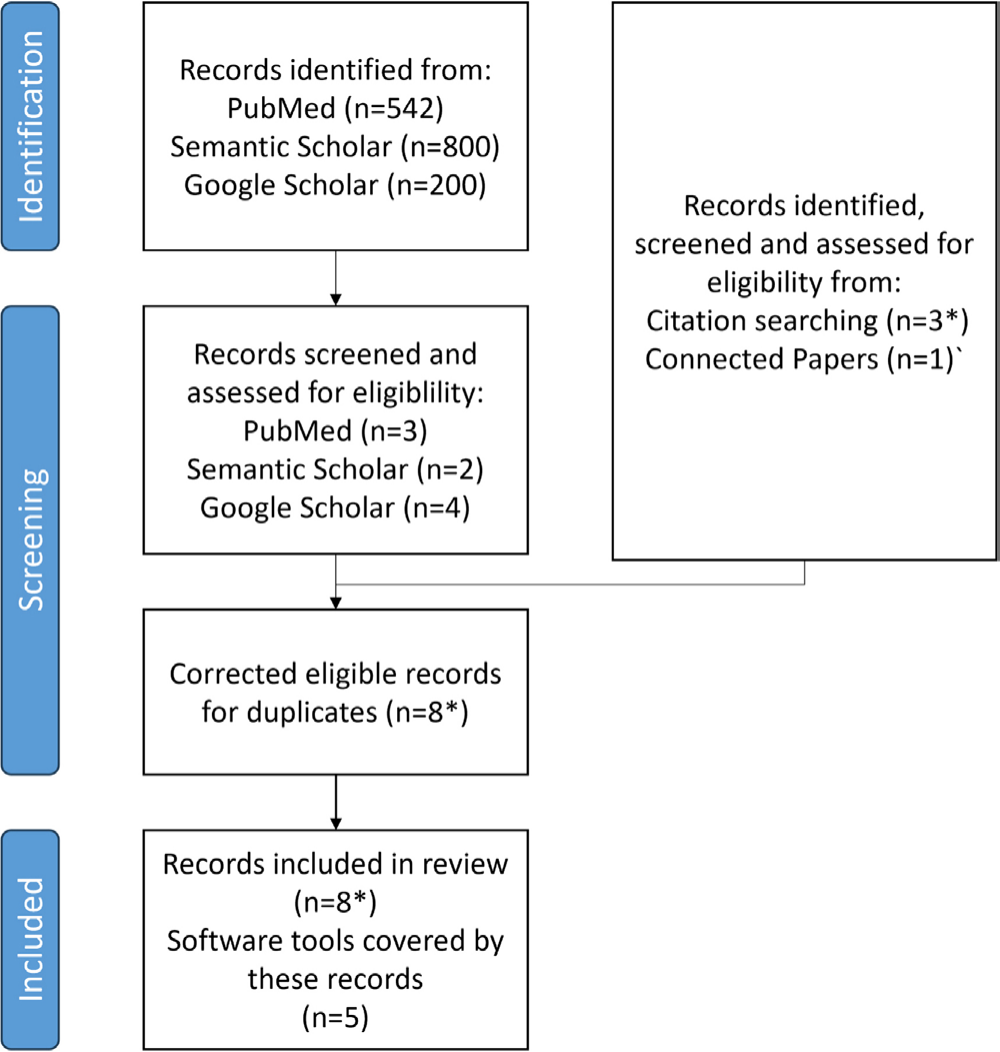
Flow chart of electronic literature search. The * denotes the presence of a reference that does not have an associated publication.

For this scoping review, we identified relevant publications using the search engines PubMed, Google Scholar, and Semantic Scholar. The following search terms were used: 1.“Multimodal brain”, 2. “Neuroimaging transcriptomics”, 3. “Imaging transcriptomics”, 4. “Brain”, 5. “Data exploration”, 6. “Data mining”, 7. “Visual analytics”. We constructed a logical statement using these search terms ((1 OR 2 OR 3 OR 4) AND (5 OR 6 OR 7)). No limitation was placed on the publication year. The first author identified eligible publications through title and abstract screening. Literature search is summarized in Figure 3. The PubMed search returned 542 results, 3 of which met the eligibility criteria defined in Section 1.1. As Semantic Scholar does not allow for Boolean search strings, four searches with different combinations of statements 1–7 were queried subsequently. Only the first 200 results of Google Scholar and each Semantic Scholar search were screened due to decreasing result quality. Of these results, two publications from Google Scholar and four publications from Semantic Scholar met the eligibility criteria. After correcting for duplicates, five publications remained. For every publication that was found eligible, reference list searching and the visual publication exploration tool were used to find additional potential candidates. In this way, four additional references could be identified, one of which does not have an associated publication. After correcting for duplicates and bundling publications describing the same software, five data exploration tools were found eligible to the criteria listed above. The identified tools were cross-checked for eligibility by two co-authors. In addition, we discussed the search results and additional, possible inclusions with neuroscientists from industry and academia. However, no additional tools were added as a result of these discussions. All tools, related publications, and web addresses for access can be taken from Table 1. We loosely followed PRISMA-ScR guidelines in the review process. A document relating PRISMA-ScR items to specific sections, figures, and tables can be found in the Supplementary Material.

**Table 1.** Results of literature search with associated publications, web addresses for access, related documentations, and the latest version/repository commit date as of October 28, 2025.

Tool	Publications	Access & Documentation	Last updated
SIIBRA Explorer	-	https://atlases.ebrains.eu/viewer/ https://siibra-explorer.readthedocs.io/en/latest/	10.2025
Brain Explorer 2	Lau et al. (2008), Feng et al. (2015)	https://mouse.brain-map.org/static/brainexplorer https://community.brain-map.org/t/allen-brain-explorer-how-to/3019	
BrainTrawler	F. Ganglberger et al. (2019, 2024)	https://braintrawler.vrvis.at/ https://braintrawler.vrvis.at/docs/index.html	9.2025
Neurosynth	Yarkoni et al. (2011), A. S. Fox et al. (2014)	https://neurosynth.org/ https://neurosynth.org/faq/	10.2025*
LinkRbrain	Mesmoudi et al. (2015)	https://linkrbrain.org/ Publication by Mesmoudi et al. (2015)	

Note that for Brain Explorer 2 and LinkRbrain, no latest dates could be found. *Development on Neurosynth was moved to the NiMARE package.

## 3 Brain Data Exploration Software

In this section, we provide a general description of all tools identified in the literature search in no particular order. A comparative evaluation can be found in the next section.

### 3.1 SIIBRA Explorer

SIIBRA (software interfaces for interacting with brain atlases) Explorer is a web-based data exploration tool provided by EBRAINS. The tool gives users access to reference atlases for the human, mouse, rat, and macaque (see Table 2) and allows them to easily find related datasets (see Tables 4 and 5).

**Fig. 4.**
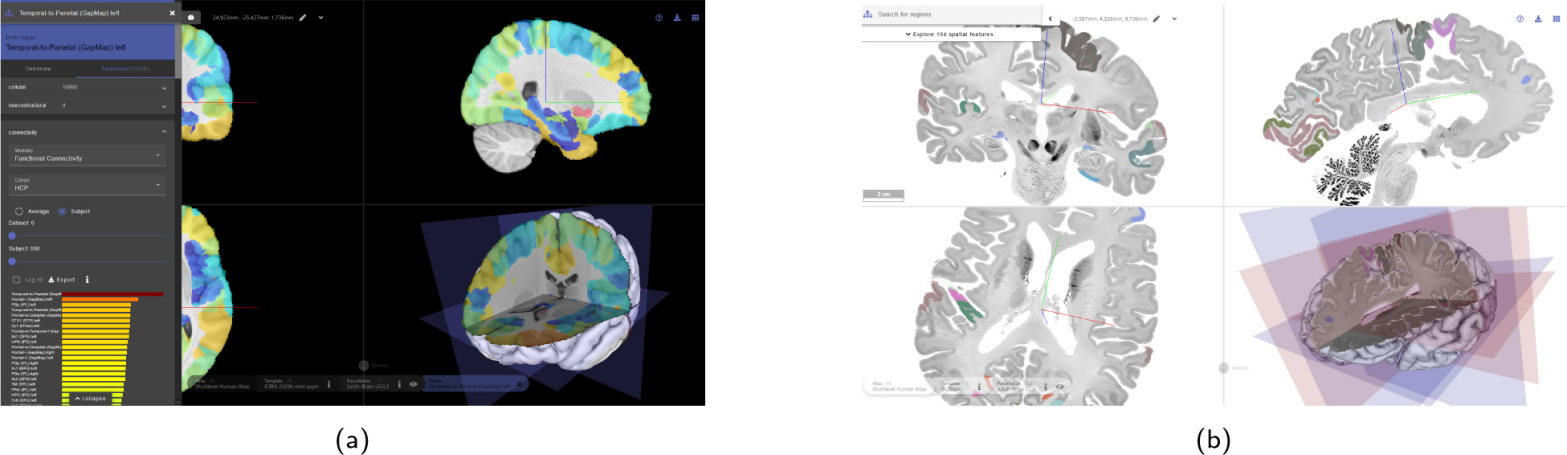
(a) Example for functional connectivity visualization in SIIBRA. On the left, a region connectivity profile is shown. (b) Oblique slicing and 3D cutaway view using the high-resolution BigBrain atlas.

Reference atlases in SIIBRA Explorer cover different facets of brain organizations by allowing multiple parcellations, for example, based on cytoarchitecture (Amunts et al., 2020), cortical layers (Wagstyl et al., 2020), white matter tracts (P. Guevara et al., 2012), and functional regions (Dadi et al., 2020). A summary of the available parcellations is given in Table 3. Many available data types, such as neurotransmitter densities, functional and structural connectivity, and cortical cell body distributions, are semantically linked to parcellation regions as “region features”. These features, as well as all templates and parcellations available in SIIBRA Explorer, can be accessed through a metadata management system called the EBRAINS Knowledge Graph (Denervaud et al., 2019). It has to be noted that not all parcellations and feature data are available for every template. For example, connectivity data and functional parcellations can be inspected with the ICBM 2009c nonlin. asym. template (see Fig. 4a), but not with the BigBrain template.

While SIIBRA Explorer focuses on letting users visually explore data, a SIIBRA Python package and an HTTP interface targeted toward reproducible data analysis workflows are also available (Dickscheid et al., 2024). Inside the application, the add-on tools JuGEx (Bludau et al., 2018) and NeurogenPy (Gutiérrez, 2022) can be used to perform differential gene expression analysis (DGEA) and construct gene regulatory networks as Bayesian networks for a desired set of genes (Delgado & Gómez-Vela, 2019), respectively. However, JuGEx can only be used with MNI-space template images.

SIIBRA Explorer facilitates visual exploration of EBRAINS templates and parcellations and is particularly useful for investigating human brain regions due to the rich EBRAINS multilevel human atlas. The BigBrain template, at 10 μm resolution (see Fig. 4b), offers the highest resolution among the tools reviewed. However, parcellations beyond cytoarchitecture and cortical layers are only supported in the MNI template. For macaque, mouse, and rat atlases, feature data are limited. Connectivity data are available for the human MNI template and rat, while transcriptomic data are accessible only for the human MNI template via the JuGEx plugin. Although SIIBRA Explorer allows for quick exploration of specific regions, it does not support explorative gene expression searches or visualizations. However, combined with JuGEx, it enables differential gene expression analysis on a brain region resolution, requiring users to specify genes of interest *a priori*.

### 3.2 Brain Explorer 2

Brain Explorer 2 is a desktop application for Windows and Mac provided by the Allen Brain Institute that can be used to explore selected transcriptomics and connectivity resources. It mainly serves as a visualization and query tool for the Allen Mouse Brain Atlas (AMBA) (Lau et al., 2008) and the Allen Mouse Brain Connectivity Atlas (AMBCA) (Feng et al., 2015). Furthermore, the tool can be used to inspect expression in the Allen Human Brain Atlas (AHBA) and the Allen Developing Mouse Brain Atlas (ADMBA). The templates, parcellations, and datasets available in Brain Explorer 2 are summarized in Tables 2 to 5.

**Fig. 5.**
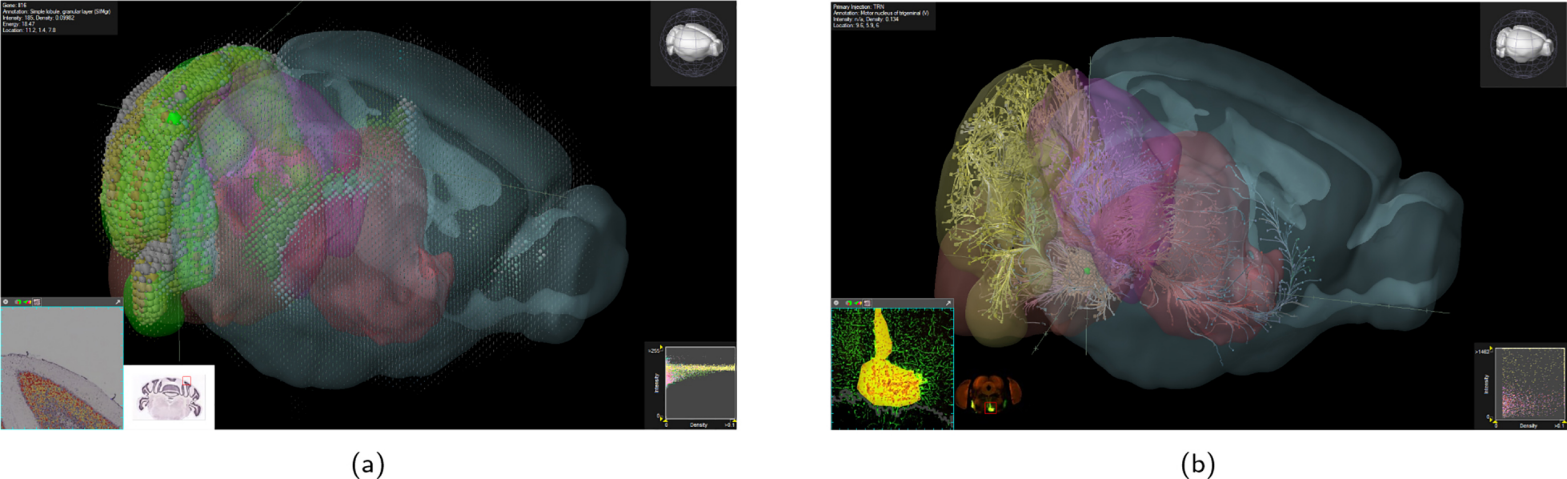
(a) Visualization of the gene expression of the gene interleukin 16 and (b) outgoing axonal projections from the tegmental reticular nucleus in Brain Explorer 2. The bottom left display shows the original (a) ISH/(b) two-photon image overlaid with the segmented signal heatmap. On the lower right, visual thresholding controls on signal intensity and density can be accessed.

In the application, users can query selected brain regions for highly expressed genes and, if available, for source (efferent) or target (afferent) axon projections. When inspecting the AMBA, a 3D gene expression visualization and the underlying high-resolution ISH slices can be inspected, as shown in Figure 5a. Spatial homology queries can be used to find genes with similar spatial expression patterns (Lau et al., 2008). Structural connectivity data from the AMBCA are visualized by marking the tracer injection site with multiple emanating tubes, depicting the projection signal, as shown in Figure 5b. All queries can also be executed on the Allen Brain website, from where results can be loaded into Brain Explorer 2. In addition, the web page allows comparing expression data between mouse and human in a differential search, but results from these queries cannot be loaded into Brain Explorer 2.

Brain Explorer 2 is designed for visual data exploration and is particularly useful for researchers studying individual genes or the structural connectivity of specific brain regions in mice. Additionally, expression in the human and developing mouse can be inspected, but no connectivity data are available for these species. Combined with the wide range of query options on the Allen Brain query system, Brain Explorer 2 offers versatile tools for searching the available transcriptomics and connectivity datasets. Specifically, it is one of the few tools that allows explorative queries for gene expression data without specifying genes of interest *a priori*. While Brain Explorer 2 offers powerful ways of querying, visualizing, and thresholding the data, the four datasets that can be explored offer little flexibility in filtering by metadata such as cell types.

### 3.3 BrainTrawler

BrainTrawler is a web-based brain data exploration tool that employs interactive visualizations and precise spatial querying. Since its introduction as an efficient tool for querying of network data (F. Ganglberger et al., 2019), BrainTrawler was extended to include multi-modal transcriptomic data (BrainTACO) (F. Ganglberger et al., 2024). The application gives users access to human and mouse data, including resting-state connectivity, axonal projections, microarray gene expression, ISH, bulk RNA sequencing, and sc/snRNA sequencing. Expression data are linked to gene ontology (GO) terms. Notably, subject age and morpho-electric cell type metadata are available for multiple scRNA seq. datasets and can be used to filter queries. Templates, parcellations, and datasets available in BrainTrawler are summarized in Tables 2 to 5.

**Fig. 6.**
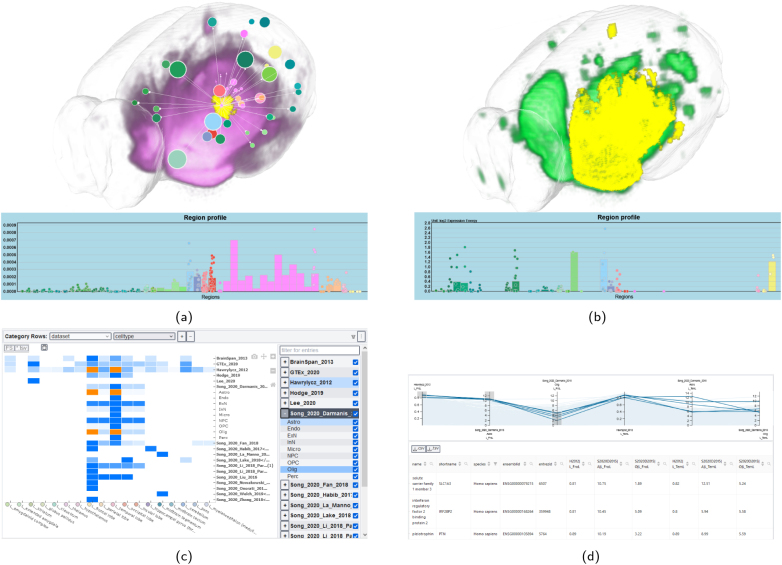
Result visualizations in BrainTrawler. (a) Target query from the VTA on axonal projection data. Outgoing connectivity is displayed as a purple cloud in 2D and 3D. Region-wise connectivity is shown via the node-and-link visualization and the region profile below. (b) Visualization of gene expression of Serpina9, obtained by creating a connectivity-based ROI from (a) by thresholding and querying the AMBA for specific expression in that ROI. (c) Heatmap visualization showing sample density across multiple datasets and brain regions. Selected dataset-cell-type-brain region combinations (tiles) in orange can be inspected in other visualizations. (d) Interactive parallel coordinates visualization showing genome-wide expression in the selected tiles. Filtering limits the gene list below the visualization.

With BrainTrawler, users can execute spatial queries on all available data, such as searching for genes specific to subsections of the amygdala. Query regions-of-interest (ROIs) are generated by drawing custom selections, selecting brain regions of interest or by applying a threshold to previous results (e.g., the outgoing connectivity of a region). Using these ROIs, target, and source queries on network data can be performed, as shown in Figure 6a. Similarly, queries for high or specific expression, as well as cell type specificity and categorical enrichment can be executed on gene expression data (see Fig. 6b). Query results are shown on a brain region level using anatomical brain parcellations. An interactive heatmap visualization is used to convey brain region coverage and sampling densities across all transcriptomic datasets. Also, genome-wide expression comparisons across different datasets and brain regions can be accessed in the application. Both visualizations are shown in Figure 6b and d.

BrainTrawler is centered around spatial querying of the available data. It provides connectivity and transcriptomics data for both mouse and human and is the only tool that allows access to scRNA seq. data, filter for different cell types, and explore results based on GO terms. Furthermore, it allows genome-wide explorative queries of gene expression data. With its broad transcriptomic database and network querying options, BrainTrawler is a useful resource for studying cell-specific expression and relationships between structural/resting-state connectivity and gene expression. However, it does not allow for differential expression queries, and the genome-wide querying interface can be confusing for users.

### 3.4 Neurosynth

Neurosynth is a web-based platform for large-scale, automated synthesis of human fMRI data that is also integrated in the Neuroimaging Meta-Analysis Research Environment (NiMARE) Python package. The application provides probabilistic mappings between cognitive ontology terms and meta-analytic activation patterns, as well as meta-analytic coactivation maps (Yarkoni et al., 2011). While its main focus is automated meta-analysis of human functional imaging data, Neurosynth also provides access to microarray expression data from the AHBA and can be used for data exploration purposes. The template and datasets provided by Neurosynth are summarized in Tables 2, 4, and 5. Neurosynth does not use brain parcellations.

**Fig. 7.**
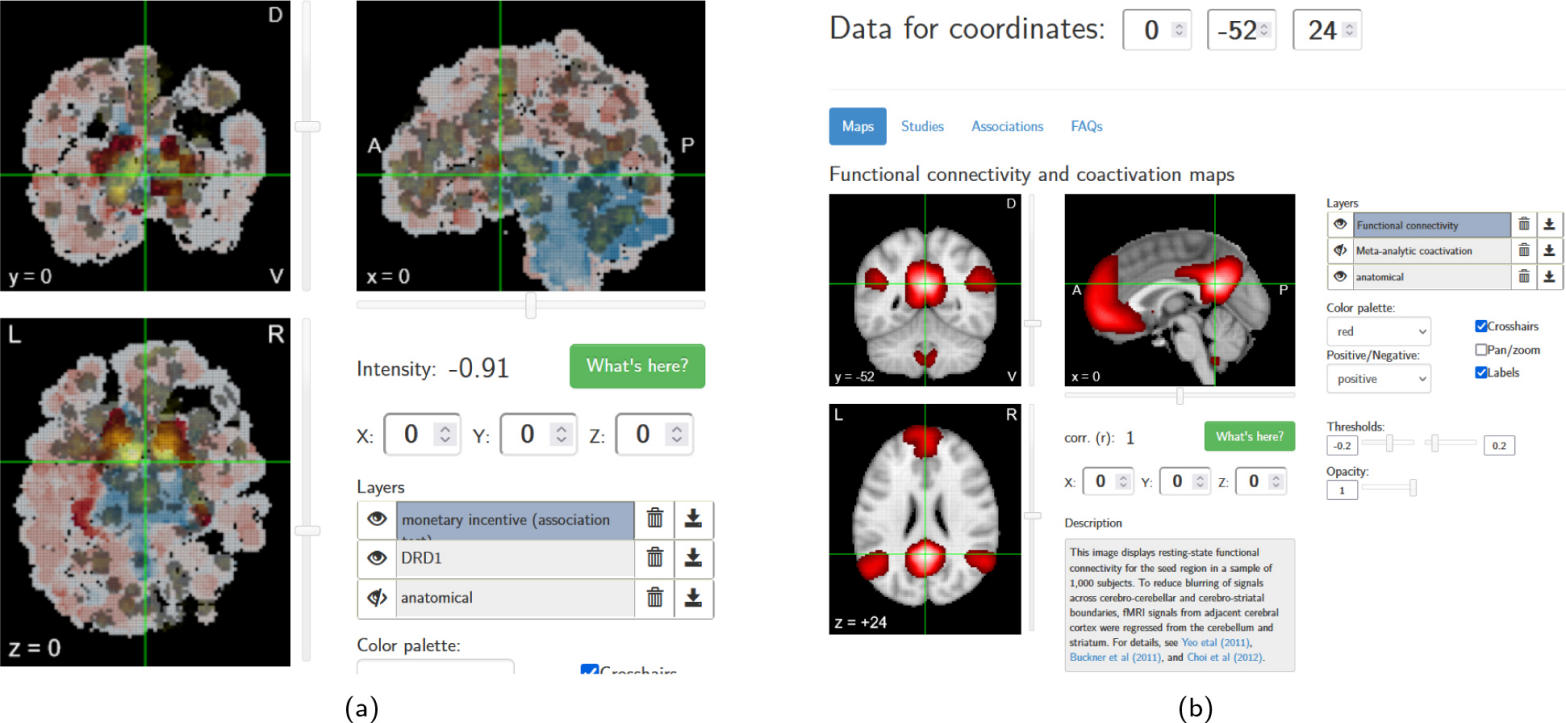
(a) Expression visualization of gene DRD1 overlaid with the meta-analytic activation map for “monetary incentive” (correlation = 0.194) in Neurosynth. (b) Functional connectivity of the seed location at the crosshair.

Neurosynth is focused on interaction with its meta-analytic (co)activation maps. On the website, users can search for cognitive terms and explore related meta-analytic activation maps. Also, AHBA gene expression patterns (Hawrylycz et al., 2012) and correlated cognitive ontology terms can be explored in the application (A. S. Fox et al., 2014) (see Fig. 7a). Using the Neurosynth image decoder, users can find associated cognitive terms for their own statistical maps. These maps can originate from arbitrary modalities such as fMRI, PET, or in-situ hybridization and must be unthresholded, in 3D NIfTI format and registered to FSL MNI standard space. To use the decoder on the web page, users must upload their maps to NeuroVault (Gorgolewski et al., 2015), a web-based public repository for statistical brain maps. In addition, Neurosynth allows users to investigate meta-analytic co-activation and resting-state functional connectivity of single coordinates, as well as related cognitive terms (see Fig. 7b).

Neurosynth is designed to provide easy access to its large database of meta-analytic functional activation data and probabilistic activation-term mappings. Especially the Neurosynth image decoder can be helpful for interpretations of neuroimaging results. In the context of imaging transcriptomics, finding cognitive terms associated with gene expression patterns allows generating hypotheses to complement existing methods such as genome-wide association studies (GWAS) (A. S. Fox et al., 2014). However, the tool was not specifically designed for imaging transcriptomics data exploration, resulting in limited query options.

### 3.5 LinkRbrain

LinkRbrain is a lightweight web-based data integration platform that combines human meta-analytic activation maps with gene expression data (Mesmoudi et al., 2015). The application integrates sensorimotor and cognitive terms and corresponding meta-analytic activation maps from Neurosynth with microarray expression from AHBA donor brain H0351.2001 (Hawrylycz et al., 2012). The template and datasets available in LinkRbrain are summarized in Tables 2, 4, and 5.

**Fig. 8.**
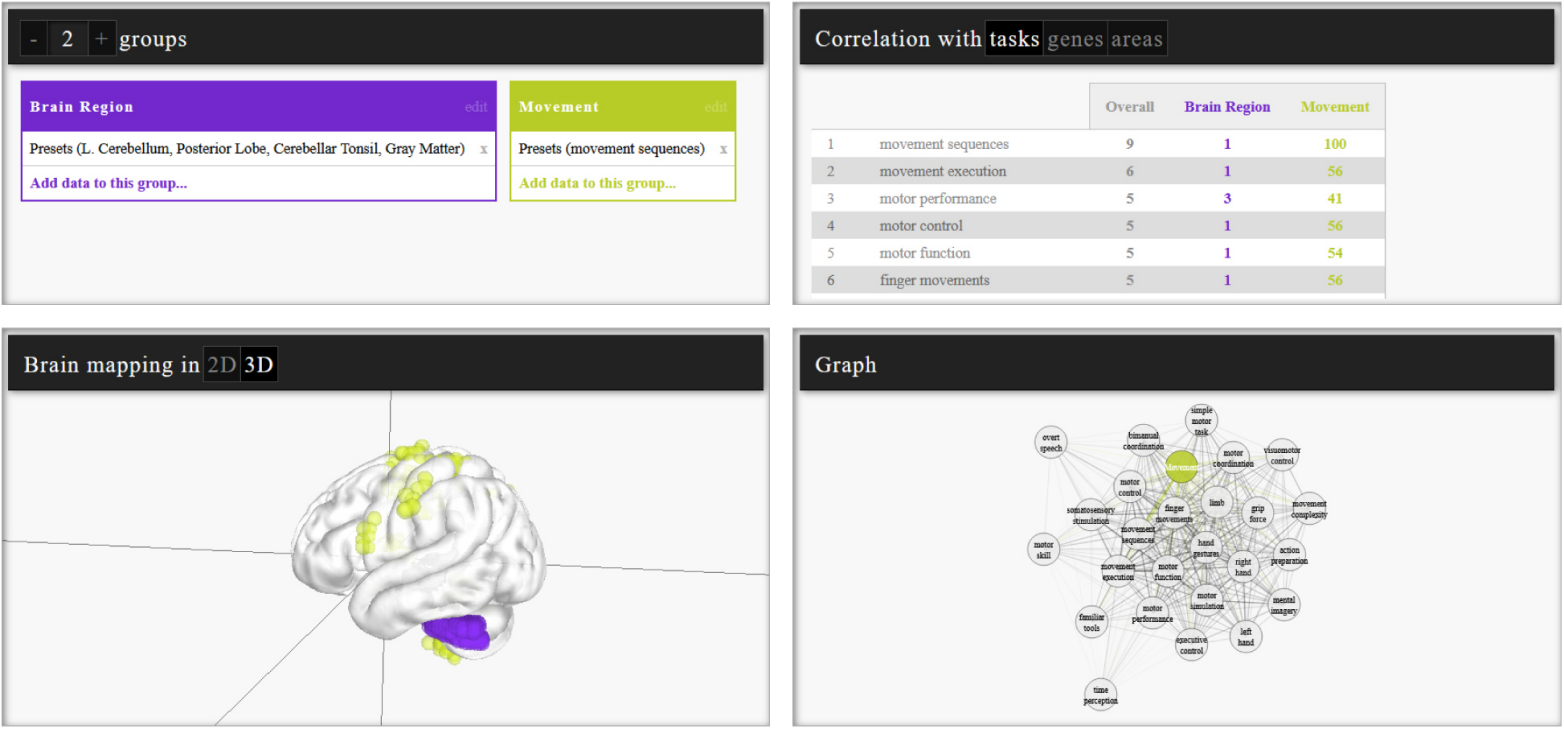
Example LinkRbrain query. The group view is visible on the top left. The purple group “Brain region” contains the left cerebellar tonsil, while the green group “Movement” contains activation coordinates related to movement sequences. A rendered brain view is available on the lower left, showing brain regions and activated regions with tasks as colored spheres. Gene expression can be visualized in the same way. A table and a proximity graph on the right display the topographical overlap (see Mesmoudi et al., 2015) of groups with different tasks, genes, or brain regions.

LinkRbrain allows users to define groups by combining genes expression patterns, task activations, and brain region locations. All coordinates related to a group are then bundled and visualized in the other panels, as shown in Figure 8. LinkRbrain uses a correlation table and a relation graph and a rendered 2D/3D brain to visualize and compare group signals. A correlative measure called “topographical overlap” is used to inform the correlation table and the relation graph. These show the overlap between the user-defined groups and single genes, task activations, or brain regions. Details for the computation of topographical overlap can be found in the original publication by Mesmoudi et al. (2015).

LinkRbrain is centered around the flexible definition of groups from different kinds of signals. It is an easy-to-use web-based tool that is suitable for the qualitative exploration of relationships between cognitive terms, regions, and genes using both the 3D viewer and the relation graph. However, LinkRbrain only provides a subset of all terms available within Neurosynth, which offers similar gene–function correlation functionalities and can also be extended with user data. Furthermore, query options for transcriptomics or connectivity data are not available.

## 4 Discussion and Evaluation

In this section, we will compare and evaluate the previously presented software. Then, we discuss the limitations of brain data exploration tools and briefly list software that can be used for subsequent imaging transcriptomics analyses.

The eligibility criteria for this review establish the fundamental requirements for data exploration tools in imaging transcriptomics research. However, their practical utility largely depends on meeting additional conditions. To provide a comprehensive evaluation, we have developed a set of key criteria against which we will assess all previously discussed software. These criteria encompass:

1. Availability of reference atlases and parcellations for multiple species
2. Access to cell type- and age-specific transcriptomics data
3. Access to task- and disease-specific neuroimaging data
4. Capability for detailed data querying and filtering based on spatial selections and metadata
5. Data export functionality
6. Capability to integrate user-generated data
7. Interactive visualization of data and results
8. Transparent data processing
9. Clear and thorough documentation
10. Active maintenance and further development

Clear motivations for each of these conditions are provided in the following sections.

### 4.1 Species, templates, and parcellations

Support for multiple species, templates, and parcellations inside an exploration tool makes it interesting to a broader audience (see criterion 1). A summary of the available species and templates in each exploration tool is given in Table 2. SIIBRA Explorer is the only exploration tool that provides templates for the rat and the macaque brain. Furthermore, it gives users access to the most template images, involving the 2D brain surface template with Freesurfer fsaverage and the most highly resolved template for the human with BigBrain. Brain Explorer 2 is unique in providing access to a time-resolved template series with the Allen Developing Mouse Brain Atlas. Neurosynth and LinkRbrain only provide data for the human. The parcellations available in each species are presented in Table 3. SIIBRA Explorer provides multiple non-hierarchical parcellations for the human brain, involving modalities such as cytoarchitecture, functional modes, and white matter fiber bundles. However, most SIIBRA parcellations are available only in the MNI 152 nonlin. 2009 template. Brain Explorer 2 and BrainTrawler use well-known hierarchical anatomical parcellations from the Allen Brain Project for human and mouse. LinkRbrain is the only tool that provides access to the Talairach atlas, which is somewhat outdated. Neurosynth does not use brain parcellations.

**Table 2.** Overview of the species and templates provided in the data exploration tools.

Species	Template	SIIBRA	Brain Explorer 2	BrainTrawler	Neurosynth	LinkRbrain
Human	MNI Colin 27 (C. J. Holmes et al., 1998)	✓				
	MNI 152 nonlin. 6th gen. (Grabner et al., 2006)				✓	✓
	MNI 152 nonlin. 2009 (Fonov et al., 2009)	✓	✓	✓		
	BigBrain (Amunts et al., 2013)	✓				
	Freesurfer fsaverage (Dale et al., 1999)	✓				
Mouse	Allen Mouse Brain CCF (Wang et al., 2020)	✓	✓	✓		
	Allen Developing Mouse Brain (Thompson et al., 2014)		✓			
Rat	Waxholm space Atlas (Papp et al., 2014)	✓				
Macaque	MEBRAINS (Balan et al., 2024)	✓				

**Table 3.** Summary of all available parcellations among the discussed tools.

Species	Parcellation	SIIBRA Explorer	Brain Explorer 2	BrainTrawler	Neurosynth	LinkRbrain
Human	Talairach Atlas (Lancaster et al., 2000)					✓
	Allen Human Reference Atlas (Ding et al., 2016)		✓	✓		
	Julich Brain Atlas (Amunts et al., 2020)	✓				
	Atlas of deep white matter fiber Bundles (P. Guevara et al., 2012)	✓^1^				
	Atlas of superficial white matter fiber bundles (M. Guevara et al., 2017)	✓^1^				
	Atlas of short fiber bundles (Avila et al., 2019)	✓^1^				
	Functional Mode Atlas (Dadi et al., 2020)	✓^1^				
	BigBrain Cortical surface (Lewis et al., 2014)	✓^1^				
	BigBrain Cortical layers (Wagstyl et al., 2020)	✓^2^				
Mouse	Allen Mouse Brain CCF (Wang et al., 2020)	✓^2^	✓	✓		
	Allen Developing Mouse Brain Atlas (Thompson et al., 2014)		✓			
Rat	Waxholm Space Atlas of the Sprague Dawley Rat Brain (Papp et al., 2014)	✓				
Macaque	MEBRAINS population-based Macaque Atlas (Balan et al., 2024)	✓				

Check marks ^1^ and ^2^ denote parcellations in SIIBRA Explorer that are only available for the MNI 152 nonlin. 2009 and BigBrain template, respectively.

### 4.2 Modalities and datasets

The available data types in a brain data exploration tool determine whether it can be used to answer certain research questions (see criteria 2 and 3). Table 4 summarizes the available transcriptomics data modalities and datasets that can be accessed and visualized with the listed software. All available connectivity and activation data modalities and datasets are listed in Table 5. While all tools give users access to microarray data from the AHBA, BrainTrawler is the only tool that also provides sc/snRNA sequencing data, allowing the study of expression in specific cell types. Developmental mouse expression data can only be inspected using Brain Explorer 2 (and the Allen Brain web page).

**Table 4.** Overview of the transcriptomic data that can be accessed and inspected with the tools discussed in this report.

Modality	Dataset	SIIBRA Explorer	Brain Explorer 2	BrainTrawler	Neurosynth	LinkRbrain
ISH	Allen Mouse Brain Atlas (Lein et al., 2006)		✓	✓		
	Allen Developing Mouse Brain Atlas (Henry & Hohmann, 2012)		✓			
Microarray	Allen Human Brain Atlas (Hawrylycz et al., 2012)	(✓)	✓	✓	✓	(✓)
Sc/snRNA seq	Linnarson Lab Adolescent Mouse Brain Atlas (Zeisel et al., 2018)			✓		
	STAB* (Nowakowski et al. (2017) and Song et al. (2021) Darmanis et al. (2015), Fan et al. (2018), and Zhong et al. (2018) Lake et al. (2017), Li and Wang (2021), and Manno et al. (2016) Habib et al. (2017) and Welch et al. (2019)			✓		
	Bhattacherjee et al. (2019)			✓		
	Yao et al. (2021)			✓		
	Campbell et al. (2017)			✓		
	Rossi et al. (2019)			✓		
	Gouwens et al. (2020)			✓		
	Gokce et al. (2016)			✓		
	Lee et al. (2020)			✓		
	Hodge et al. (2019)			✓		
Bulk RNA seq	BrainSpan Consortium (2011)			✓		

Note that microarray data in SIIBRA can only be accessed via the JuGEx plugin. LinkRbrain provides only cortical samples from a single donor brain of the AHBA (Mesmoudi et al., 2015). *Excluding STAB-datasets h12–h14 (see Song et al., 2021).

**Table 5.** Overview of the connectivity data and activation data that can be accessed and inspected with the tools discussed in this report.

Modality	Dataset	SIIBRA Explorer	Brain Explorer 2	BrainTrawler	Neurosynth	LinkRbrain
DTI	HCP S1200 Young Adult (“Human Connectome Project”, 2023)	✓				
	CHENONCEAU (Beaujoin et al., 2019)	✓				
Tract tracing	Allen Mouse Brain Connectivity Atlas (Oh et al., 2014)		✓	✓		
	Reiten and Leergaard (2023)	✓				
Resting-state fMRI	HCP S1200 Young Adult (“Human Connectome Project”, 2023)	✓		✓		
	Brain Genomics Superstruct Project (A. J. Holmes et al., 2015)				✓	
Meta-analytic activation	Neurosynth (Yarkoni et al., 2011)				✓	✓

Brain Explorer allows users to access individual injection experiments, BrainTrawler aggregates all injection experiments into one connectivity matrix that can be queried (see supplementary note 1 in F. J. Ganglberger et al., 2020).

It is noteworthy that transcriptomics data are provided on different levels of detail and/or aggregation levels across applications, making a direct comparison of results challenging. We provide an overview of the used pre-processing steps and aggregation levels in Table 6 in the most relevant transcriptomics-related query of each tool. In Brain Explorer 2, expression queries return lists of probes, meaning that genes can occur multiple times in the result. In contrast, the other applications use different pre-processing and aggregation strategies to handle probes of the same genes. During the execution of differential expression queries with JuGEx (SIIBRA Explorer), the expression of multiple probes is aggregated by taking the winsorized (10–90%) average of all probes of a single gene (Bludau et al., 2018). During pre-processing, Neurosynth averages all probes of each sample site for each donor and gene, then normalizes the expression across sample sites and averages over all donors. For each gene, this results in a list of expression values at each sample site, which is then integrated (A. S. Fox et al., 2014). In BrainTrawler, sample data are pre-aggregated in the lowest-level parcellation regions. During queries, the average of all pre-aggregated samples in all brain regions inside the query volume is computed to produce a single expression value per gene (F. Ganglberger et al., 2024). LinkRbrain provides the cortical probes of a single AHBA donor brain and averages over probes of the same gene before queries are executed (Mesmoudi et al., 2015).

**Table 6.** Overview of the central transcriptomics queries in each tool with the involved preprocessing and aggregation strategies, using the AHBA dataset as an example.

Example: Allen Human Brain Atlas 3,702 sample locations covering all major neuroanatomical structures (cortical and subcortical), ∼ 20,000 protein-coding genes measured in 58,692 probes taken from 6 donor brains				
Query type	Tool	Transcriptomics preprocessing	Query aggregation	Result
Gene–term association	Neurosynth	Average probes for each sample location, donor, and gene. Normalize across sample locations for each donor and gene. Then, average over all donors.	None	Gene–term correlation
	LinkRbrain	Only cortical samples of donor H0351.2001. Average over probes for each sample location and gene	None	Topographical overlap between genes, terms, and brain regions
Spatial expression query	Brain Explorer 2	None	None	Probes with high or specific expression in brain region. Differential expression in probes using t-test.
	SIIBRA Explorer (JuGEx)	Normalized expression values across sample sites for each donor (provided by Allen Brain).	Winsorized (10–90%) average of probes in chosen parcellation regions for selected gene	Differential expression of single gene across brain regions using ANOVA.
	BrainTrawler	Normalize expression values with outlier-robust sigmoid function across sample sites for each donor. Pre-aggregation of samples in same lowest-level brain region.	Average of pre-aggregated samples in brain regions inside ROI for each gene	Genes with high or specific expression in ROI. Specific expression using fold-change to brain.

The operations listed under “Transcriptomics preprocessing” are done once when integrating the data, while the operations in “query aggregation” are performed at runtime.

In addition, a multitude of connectivity data can be accessed through SIIBRA, and a wide range of functional and structural parcellations is available. Notably, LinkRbrain and Neurosynth are the only tools that allow users to study brain activation patterns.

### 4.3 Querying and filtering

Interactive queries and filtering options are essential tools for users navigating large datasets, and often serve as a preliminary step before conducting further analyses (see criterion 4). The nature of these queries varies between neuroimaging and transcriptomics data due to the distinct structural differences in the underlying datasets.

Brain Explorer 2 and BrainTrawler both support genome-wide queries for identifying high or specific expression levels within a parcellation region. In Brain Explorer 2, results are returned on a probe level, while BrainTrawler aggregates over probes and provides gene-level results (see Table 6). BrainTrawler extends this capability by allowing arbitrary spatial selections through signal thresholding and manual region-of-interest (ROI) definition. Additionally, BrainTrawler enables users to query gene expression across multiple datasets and brain regions (see Fig. 6c and d). Both applications provide functionalities for sorting and filtering the resulting gene tables. While all applications, except SIIBRA Explorer, support queries for single gene expression, SIIBRA Explorer offers access to gene data only through the JuGEx plugin. There, users can execute differential expression queries, comparing the expression of single genes between parcellation regions. Brain Explorer 2, BrainTrawler, and Neurosynth allow interactive thresholding of single gene expression data to find sites of high/low expression.

When it comes to querying functional or structural connectivity, all applications except LinkRbrain offer this feature. LinkRbrain is limited to providing meta-analytic activation maps. In Neurosynth, connectivity queries are restricted to a single seed coordinate, whereas SIIBRA Explorer and Brain Explorer 2 allow queries for any parcellation region, and BrainTrawler supports arbitrary ROIs. The resulting connectivity data can be filtered and thresholded in BrainTrawler, Brain Explorer 2, and Neurosynth. Additionally, BrainTrawler allows querying the overlap between two networks.

A direct, quantitative comparison of query results across all discussed tools is not possible, since there is no query type that is available for all of them. Even comparison between tools with similar queries and data (e.g., genome-wide queries of the AHBA dataset in Brain Explorer 2 and BrainTrawler) is not directly possible, as the tools use different transcriptomics preprocessing and aggregation strategies and return different results (see Table 6).

### 4.4 Data export and extensibility

In the realm of data exploration software, the ability to export data after querying and filtering is a significant advantage, as it alleviates the need for researchers to develop custom data processing scripts for further analysis (see criterion 5). The second row in Table 7 highlights the tools that support data export functionalities. For instance, SIIBRA Explorer facilitates the download of regional feature data directly from its web interface, while parcellation data can be accessed via the EBRAINS knowledge graph. Brain Explorer 2 offers data downloads from the Allen Brain web page, and BrainTrawler supports the export of gene expression result lists, networks, and expression/connectivity profiles. Neurosynth provides the capability to download activation maps as NIfTI files. In LinkRbrain, users can request a comprehensive result report that includes standard brain views, relational graphs, correlation tables, and references to data sources.

**Table 7.** Overview data import and export capabilities of the tools.

	SIIBRA Explorer	Brain Explorer 2	BrainTrawler	Neurosynth	LinkRbrain
Data Export	✓	✓	✓	✓	(✓)
User Data Import	(✓)		(✓)	✓	

LinkRbrain can generate result PDF files, but these are harder to further process than conventional data formats such as csv. The user data integration in BrainTrawler and SIIBRA Explorer is experimental and not quite user friendly.

The versatility of exploration software is notably enhanced by the capacity to import, integrate, and analyze user-provided data (see criterion 6). The third row in Table 7 indicates which tools can be extended with user data. SIIBRA Explorer allows users to overlay their data in the viewer as an experimental feature. Moreover, the SIIBRA Python package and HTTP interface accept brain activity maps to identify corresponding brain regions within various parcellations, ensuring that user data are processed locally without server uploads. Conversely, the BrainTrawler web application lacks support for user data integration, though users can run the application locally using publicly available compiled files and processing scripts provided by F. Ganglberger et al. (2024). Neurosynth enables users to perform image decoding with their statistical maps in 3D NIfTI format and FSL MNI standard space. To utilize the Neurosynth image decoder as a web service, users must upload their maps to NeuroVault (Gorgolewski et al., 2015), a secure web-based repository for statistical brain maps. Alternatively, the NiMARE package allows for image decoding without the need for data uploads.

### 4.5 Data visualization

One of the most promising approaches to enable humans to understand complex data is to develop (interactive) visualizations that leverage the extremely effective visual perception of the human brain (Goldstone et al., 2015) (see criterion 7). The tools considered in this report highlight different aspects of the same data by providing different visualization types.

**Fig. 9.**
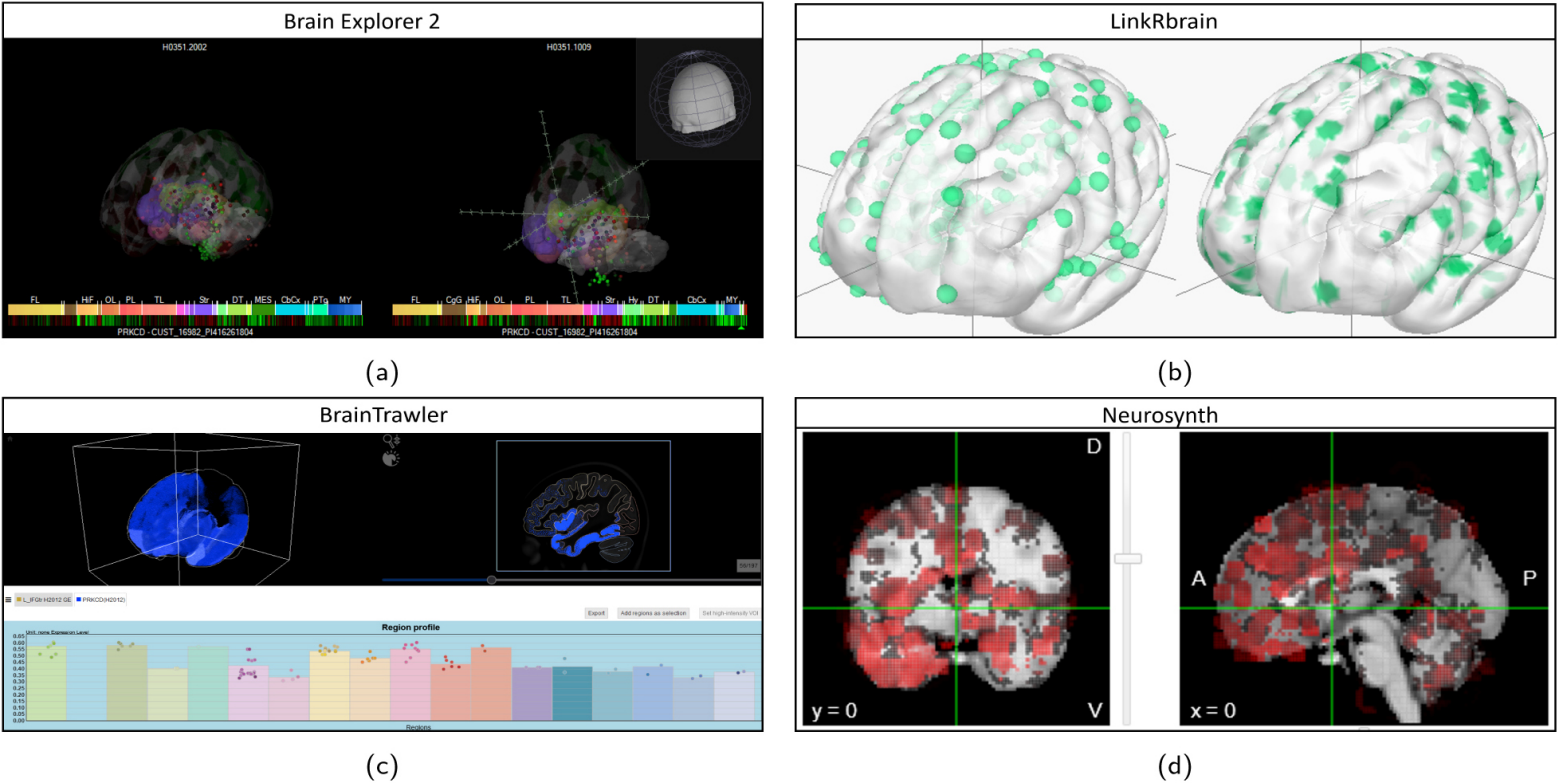
Example expression visualizations of gene PRKCD from the AHBA. (a) Brain Explorer 2 shows the expression across multiple donor brains in 3D using spherical glyphs, where color denotes expression z-score. (b) LinkRbrain visualizes expression in sample sites from a single donor brain using spherical glyph size (left) or projection onto the cortical surface (right). (c) BrainTrawler shows aggregated expression across brain regions in 3D, 2D, and with a region profile. (d) Neurosynth uses spherical glyphs, where color denotes expression z-score in 2D slices.

**Fig. 10.**
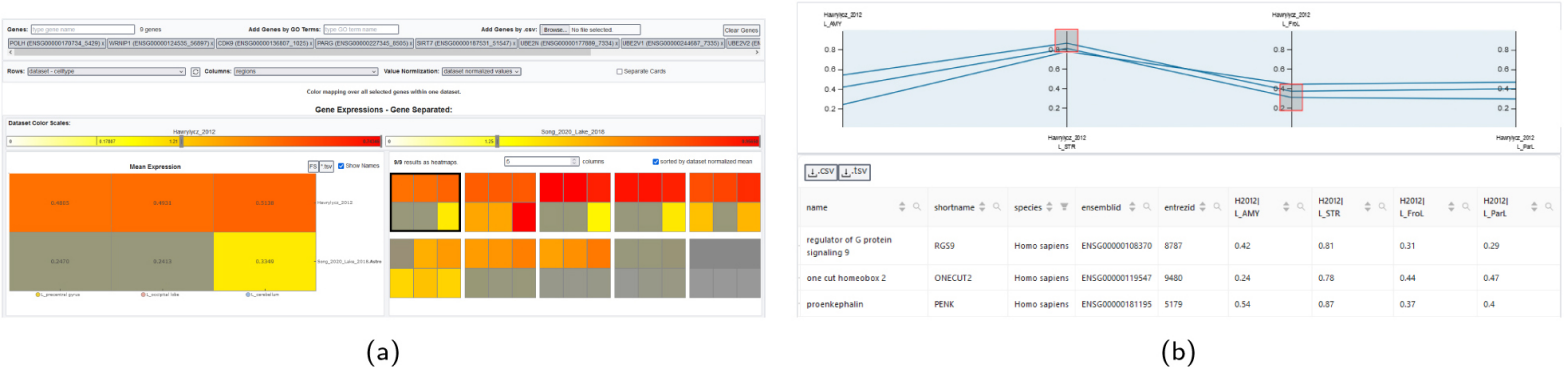
Expression visualization of multiple genes in BrainTrawler. (a) Heatmaps visualizing expression in a set of genes for a selection of datasets and brain regions. Single gene expression for each selected tile is shown on the lower right, with a detailed view on the lower left. Thresholding controls on the colorbar allow filtering. (b) Genome-wide expression visualization for a selection of datasets and brain regions using interactive parallel coordinates. Red boxes indicate filtering conditions for regional expression. Genes meeting these conditions are shown as dark blue lines and are listed in a table below.

The available types of single-gene expression visualization are shown in Figure 9 for AHBA data. Brain Explorer 2, LinkRbrain, and Neurosynth each use spherical glyphs located at the tissue sample sites to visualize expression (see Fig. 9a, b and d). This approach conserves the exact sample location. BrainTrawler aggregates tissue sample data into brain regions and visualizes the mean expression across aggregated samples in 3D, 2D, and in a region profile (see Fig. 9c). This visualization highlights expression distribution across brain regions, but omits sample location and their expression variability. BrainTrawler is currently the only tool that provides concurrent expression visualizations for large number of genes, as shown in Figure 10a and b. It allows users to inspect the expression of gene sets on a brain region level using an interactive heatmap visualization, and also enables investigating genome-wide expression on a brain region level using parallel coordinates.

**Fig. 11.**
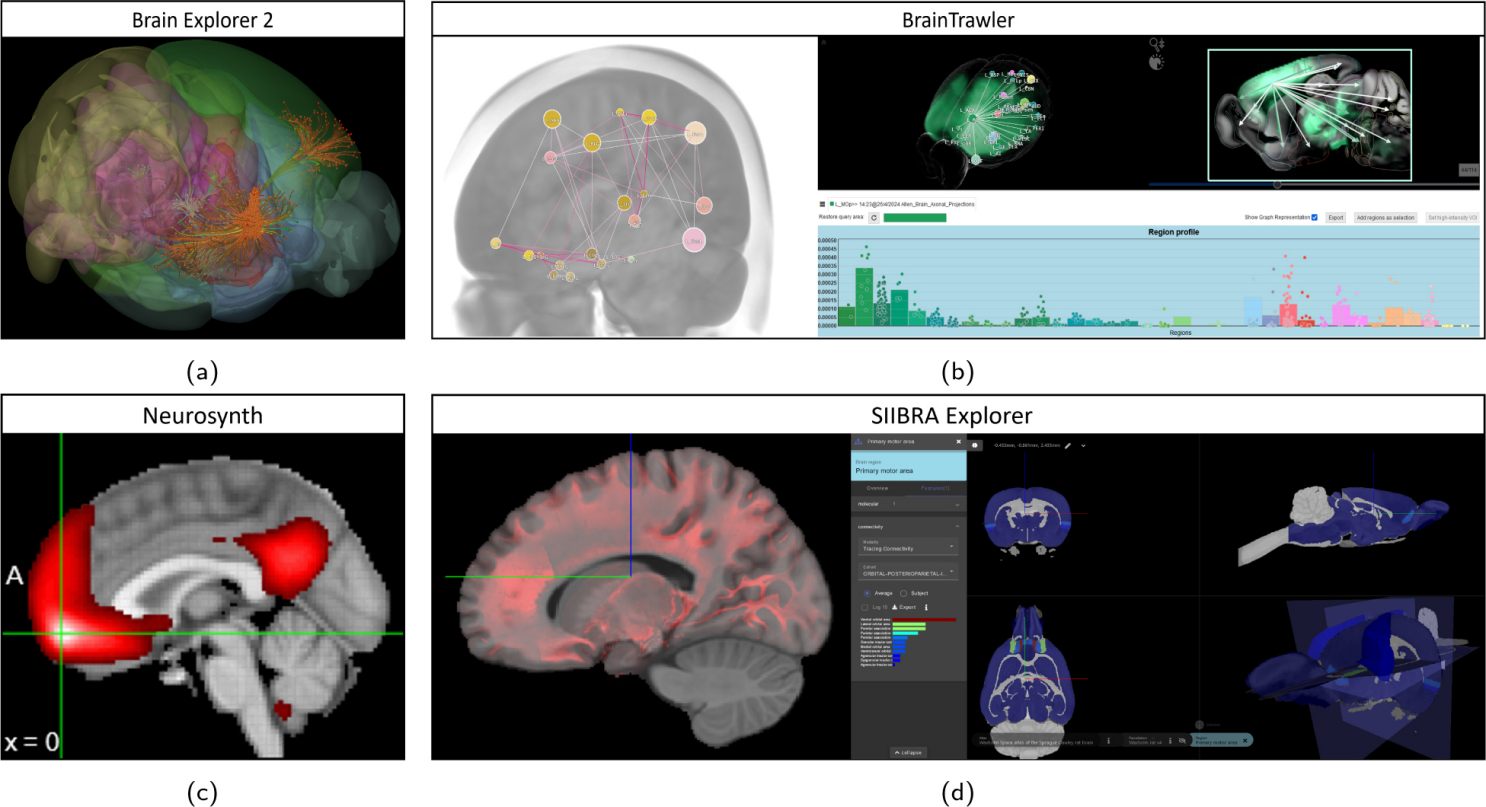
Example connectivity data visualizations. (a) 3D reconstructed axon tracts in Brain Explorer 2 for a single tracer experiment. Color denotes signal intensity. (b, left) Node-link network visualization in BrainTrawler. Connectivity strength is denoted by line color. (b, right) Voxel-wise outgoing connectivity in 2D and 3D in BrainTrawler. A superimposed node-link visualization and a bar chart capture regional connectivity. (c) Sagittal view showing the functional connectivity of a seed voxel in Neurosynth. (d, left) Sagittal view of DTI fractional anisotropy superimposed on a template image in SIIBRA Explorer. (d, right) Outgoing regional connectivity in 2D, 3D, and as a bar chart in SIIBRA Explorer. Color denotes connectivity.

Intuitive visualizations for dense network data are challenging, since they have to deal with issues such as clutter and occlusion (Sporns, 2011). To circumvent such issues, visualizations often only show a subset of the network, for example, the incoming/outgoing connectivity of a seed voxel or region. For a detailed discussion of macroscopic brain network visualizations, we refer to Chen et al. (2018). Connectivity data visualizations available with the considered tools are summarized in Figure 11. Brain Explorer 2 (Fig. 11a) shows reconstructed outgoing axonal tracts for viral tracing injection site. This visualization is useful when studying the connectivity of specific locations, but does not quantify the connectivity to other brain regions. Interactive node-link visualizations in BrainTrawler (Fig. 11b, left) allow users to inspect entire networks on a brain region resolution, but do not communicate connection strength clearly. 3D and 2D renderings of ingoing/outgoing connectivity of a selection of voxels (Fig. 11b, right), a single brain region (Fig. 11d, right), and a single seed voxel (Fig. 11c) are shown in BrainTrawler, SIIBRA, and Neurosynth, respectively. BrainTrawler and SIIBRA Explorer also use bar charts to quantify region-wise connectivity (see Figure. 11b and d). These visualizations are useful for studying regions in isolation, but do not account for secondary connections of the inspected region. Finally, 3D images capturing connectivity can also be used for visualization, as shown for a DTI FA map (Fig. 11d, left) in SIIBRA Explorer. This image superposition allows finding spatial similarities between anatomy and fiber density, but does not provide quantitative information about connectivity.

Abstract visualizations of similarity between spatial expression or activation pattern enable the user to compare multiple patterns at a glance by using distance metrics. In LinkRbrain, a graph visualization allows users to explore the topographical overlap (i.e., correlation) between custom groups and somatosensory-related terms, brain regions, and gene expression patterns (see bottom right in Fig. 8). This kind of visualization enables high-level comparisons of patterns without facing common 3D visualization problems such as occlusion and clutter. To investigate the spatial nature of these correlations in detail, other visualizations must be used.

### 4.6 Data integration approaches

It is important to understand the data integration methods used in each software to be able to correctly interpret the visualized data and query results (see criterion 8). The available data resolution and integration approaches are summarized in Table 8. For a summary of the listed data types, we refer to Section 2.3. Notably, dense data are supported by all tools, while only SIIBRA Explorer and BrainTrawler integrate semantic data. Neurosynth explicitly converts sparse AHBA data to dense data by assigning sample values to a 6 mm hard sphere around each sample point (A. S. Fox et al., 2014). Brain Explorer 2 and LinkRbrain use sparse expression data directly for voxel value assignment and use spherical volumes for visualization purposes only.

**Table 8.** Data integration approaches employed by the discussed software.

	Dense data	Sparse data	Semantic data
SIIBRA Explorer	Resampling	Aggregation to semantic, Direct (JuGEx)	Mapping to dense
Brain Explorer 2	Resampling	Direct	-
BrainTrawler	Resampling	Aggregation to semantic	Mapping to dense
NeuroSynth	Resampling	Spherical volume assignment	-
LinkRbrain	Resampling	Direct	-

The SIIBRA JuGEx plugin directly uses sparse AHBA data for computations. For explanations of the integration methods, see Section 2.3.

### 4.7 Documentation and processing transparency

Thorough software documentation and workflow transparency make tools more accessible and allow users to find and understand features effectively (see criteria 8 and 9). Well-written documentation clearly states details about data processing, internal computations, and explains results shown to the user. BrainTrawler provides a clear, web-based documentation for most UI elements, query computations, and basic workflows. A description of the pre-processing of datasets can be found in the associated publication (F. Ganglberger et al., 2024). Neurosynth handles documentation by providing embedded query descriptions and multiple FAQ sections explaining commonly asked questions. In combination with the related publication (Yarkoni et al., 2011), data processing can be understood clearly. The documentation for Brain Explorer 2 was recently moved into the Allen Brain Map Community Forum as a series of posts, which makes it slightly harder to find specific information compared with regular manuals. However, it is the only form of documentation that allows for user participation via comments. Data processing for all data in Brain Explorer 2 is explained in the related publications and on the Allen Brain web page. In SIIBRA Explorer, a small help field inside the application is combined with a more detailed documentation page, but information on queries using plugins is not provided. Information on the processing of each dataset can be found in the corresponding EBRAINS Knowledge Graph entry. Finally, the LinkRbrain web page offers a brief explanation of the underlying concepts. Details for data processing and computations can be found in the related publication (Mesmoudi et al., 2015). Links to the software tools and corresponding documentation are given in Table 1.

### 4.8 Maintenance and further development

In order to ensure long-term functionality, software needs to be actively maintained. This is especially true for web-based applications, where changing web standards or outdated dependencies can lead to unexpected problems. We contacted the developers of all investigated tools and found that BrainTrawler, SIIBRA Explorer, and LinkRbrain are being actively maintained. The development team of BrainTrawler is planning a new feature release in late 2025. LinkRbrain is now being developed and maintained as part of the LinkRdata platform, which operates as a subscription-based model. However, no new version will be published on the freely available platform. In contrast, development on Brain Explorer 2 and Neurosynth has been stopped. In 2020, the Allen Brain Institute released a statement about retiring the Brain Explorer 2 desktop application and replacing it with a new, browser-based 3D viewer titled “Brain Explorer beta”. This application offers similar features for visualizing and exploring mouse brain connectivity than the desktop application, but is limited to mouse connectivity data and does not offer support for additional data modalities or species. For Neurosynth, a follow-up platform called Neurosynth Compose has been released, which provides advanced meta-analysis features, but does not provide any functionalities related to transcriptomics data.

### 4.9 Example workflows with existing platforms

In this section, we highlight the capabilities and constraints of the previously described tools by applying them to answer example exploratory questions to brain datasets with increasing granularity. These questions were collected in collaboration with domain experts listed in the acknowledgments and aim to inform users in their choice of software.

#### 4.9.1 Which regions are functionally/structurally connected to region Y?

Often, functionally and structurally connected regions are of interest when investigating the consequences of treatment in a single region. An example is the search for the correct stimulus location to target deep brain regions with transcranial magnetic stimulation (Cash et al., 2021). Target and source queries on structural connectivity can be performed in SIIBRA Explorer, Brain Explorer 2, and BrainTrawler. Functional connectivity can be investigated in SIIBRA Explorer, BrainTrawler, and Neurosynth. These tools differ in the allowed ROIs for queries. SIIBRA Explorer and Brain Explorer 2 use brain regions as ROIs, while BrainTrawler allows for voxel-wise querying of connectivity. In Neurosynth, functional connectivity is queried for single coordinates. For this workflow, we recommend SIIBRA Explorer if the user is interested in investigating connectivity in a wide range of parcellations and BrainTrawler if the user is interested in querying precise spatial ROIs.

#### 4.9.2 Where is gene X expressed?

The expression of functionally annotated genes, such as oxytocin, can help to relate these functions to spatial locations in the brain (Quintana et al., 2019). Brain Explorer 2, BrainTrawler, Neurosynth, and LinkRbrain allow users to investigate single gene expression patterns. BrainTrawler allows expression comparison among multiple datasets per species and gives users access to scRNA expression data. In contrast, Brain Explorer 2, Neurosynth, and LinkRbrain only allow users to inspect expression based on a single dataset per species. For this workflow, we recommend using BrainTrawler to be able to cross check results using different datasets. While single gene expression patterns cannot be accessed using SIIBRA Explorer, users can perform human differential expression analysis between two regions on a set of target genes using the JuGEx plugin.

**Fig. 12.**
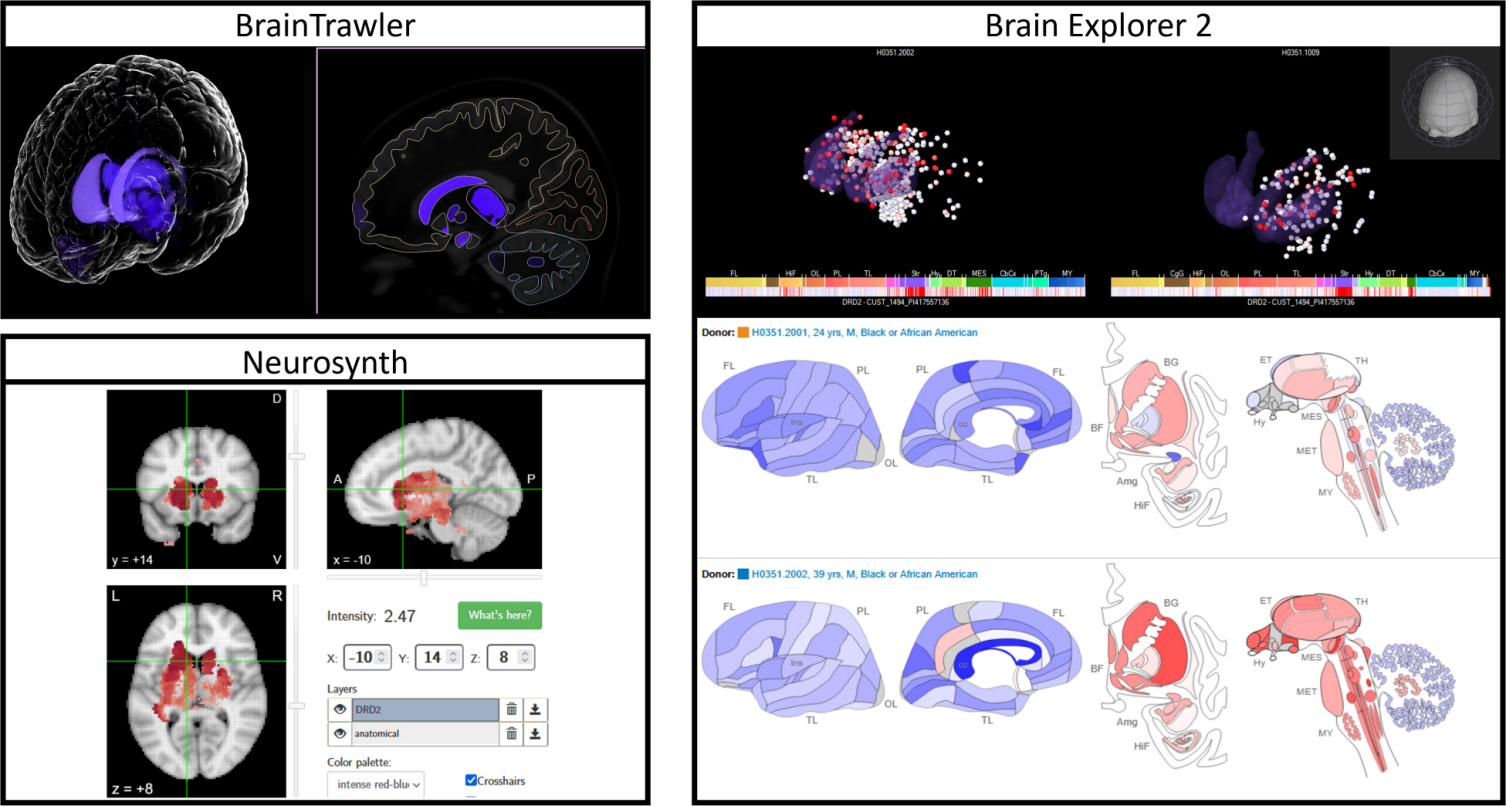
Visible-specific expression of gene DRD2 in the basal ganglion, shown in BrainTrawler, Brain Explorer 2, and Neurosynth.

To highlight differences between the described tools, we investigated whether a consistent expression pattern of a set of predefined genes from the AHBA can be investigated using all tools. For this, we explored the expression of genes specific to the basal ganglia (DRD2; Hall et al., 2003) and occipital lobe (SCN1B, SYT2, KCNA1; Hawrylycz et al., 2012). Specific expression patterns could only be observed using Brain Explorer 2, BrainTrawler, and Neurosynth, as indicated in Figure 12. While LinkRbrain does provide 2D and 3D visualizations of expression, only cortical expression data are available and visual encoding of expression values is not optimized to highlight specific patterns.

**Fig. 13.**
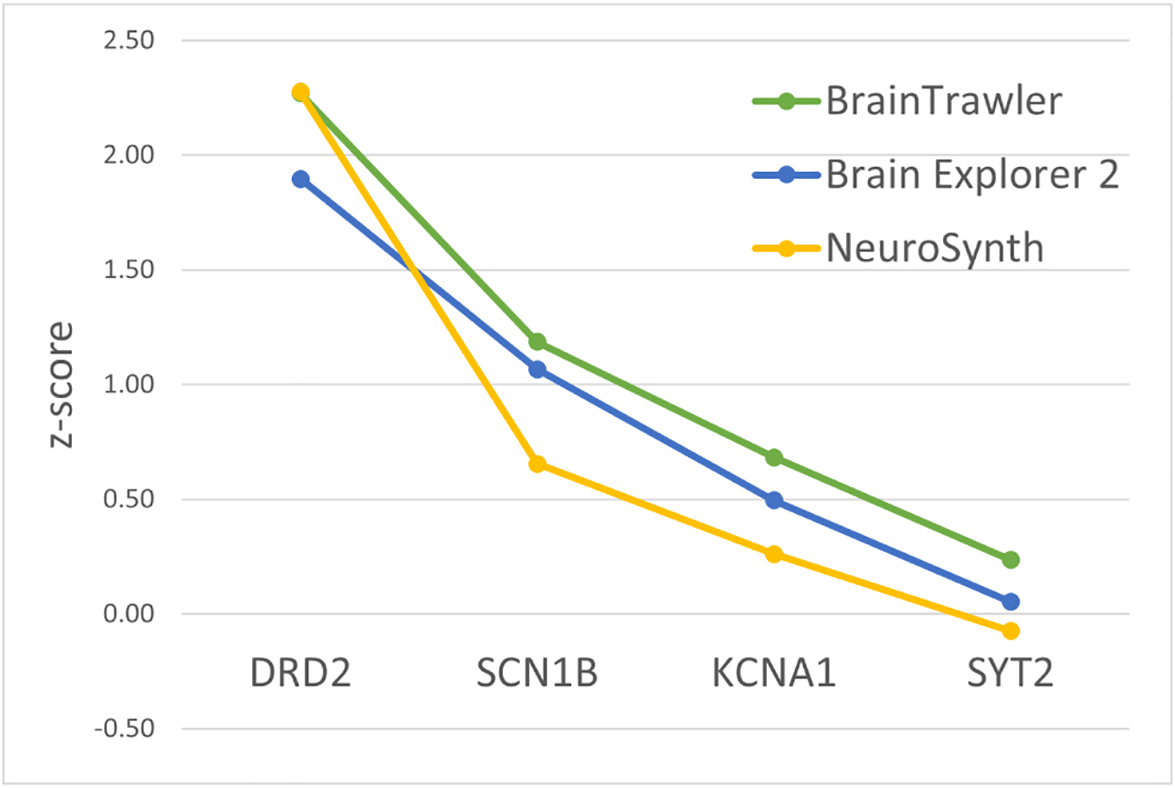
Comparison of expression z-scores in a set of marker genes across multiple tools. Quantitative expression data cannot be obtained in LinkRbrain or SIIBRA Explorer. The data used for this comparison are provided on Zenodo (https://doi.org/10.5281/zenodo.17973995).

The direct extraction of a region-wide z-score was not possible in any tool. Whenever possible, we derived z-scores of the expression values in regions of specific expression, as shown in Figure 13. However, due to the significant differences in probe aggregation, preprocessing, and data export possibilities, deviations across the results obtained from the different tools can be expected in this comparison. For calculating z-scores in BrainTrawler, we exported the region profile of each investigated gene and compared the expression across all available regions. For Brain Explorer 2, we used the Allen Brain webpage to download the expression data for all available probes for each gene. We then averaged the reported z-score over all samples in the target region and took the average across all probes to obtain a single z-score per gene. While Neurosynth does show z-score maps of expression, we had to manually sample coordinates in the target regions to obtain an average z-score, which limits the number of genes we were able to compare with reasonable effort. The required data and a detailed description of this comparison can be obtained from Zenodo (https://doi.org/10.5281/zenodo.17973995).

The differences in z-score visible from Figure 13 can be attributed to multiple factors. First, the investigated tools use different preprocessing mechanisms for genetic data, as also discussed in Section 4.3. While Brain Explorer 2 (or more specifically, the Allen Brain webpage) provides z-score data for all probes of a single gene, BrainTrawler employs outlier-robust normalization (Arnatkeviciute et al., 2019) before aggregating probe data in the smallest possible parcellation region (F. Ganglberger et al., 2024). Neurosynth uses a slightly different preprocessing workflow, averaging probes for each sample site, donor, and gene, then normalizing across sample locations and finally averaging over all donors to obtain a single expression z-score at each sample location, for each gene. In addition to differences in data processing, the three investigated tools provide results on different levels of (spatial) resolution. While Brain Explorer 2 allows the export of all probe expression data for a gene, BrainTrawler provides only the average expression across multiple regions, which introduces an uncertainty into the z-score computation. Furthermore, since Neurosynth only allows the retrieval of expression data for one coordinate point at a time, our sampling-based approach is also bound to introduce uncertainty. Nevertheless, we were able to observe similar trends using all three compared tools, both in visualizations and in a rough quantitative comparison. However, due to the considerable effort involved in manual sampling for obtaining z-score averages in Neurosynth, we recommend using either Brain Explorer 2 or BrainTrawler for this task.

#### 4.9.3 How does the expression of gene X change across different species?

By investigating the variation in gene expression among species, researchers can form hypotheses about the evolutionary origins of cell types and functions (Whitehead & Crawford, 2006). Inter-species comparisons of gene expression are currently only possible by using the Human differential queries on the Allen Brain website. These queries enable side-by-side viewing and comparison of differential search results across the mouse ISH (Lein et al., 2006) and human microarray (Hawrylycz et al., 2012) datasets, using the NCBI HomoloGene data to link genes across organisms.

#### 4.9.4 How does the expression of gene X change across different developmental stages?

Together with environmental input, coordinated, transient gene expression establishes the primary patterns in brain connectivity during development (Stiles & Jernigan, 2010). For the mouse, the Allen Developing Mouse Brain Atlas (Henry & Hohmann, 2012) can be queried in great detail on the Allen Brain web page. It provides ISH gene expression data across eight developmental stages, with subject age ranging from 11.5 days (E11.5) to 8 weeks (P56). These data can then be loaded in Brain Explorer 2, where it is superimposed on a series of eight different template images, each corresponding to one developmental stage. BrainTrawler also provides age-annotated RNA seq datasets for mice and human, specifically the dataset from Bhattacherjee et al. (2019) for mouse and the BrainSpan Consortium (2011) and the included STAB datasets (Song et al., 2021) (see Table 4) for human. Gene expression can be limited with age annotation filters, which can be further combined with other metadata filters, such as cell types. However, side-by-side comparisons of different developmental templates are not possible in BrainTrawler.

#### 4.9.5 Which genes are highly expressed in region Y?

Neurodevelopmental disorders are associated with alterations in brain anatomy. The expression of genes with copy number variations was found to correlate with these alterations by closely investigating expression in regions with altered anatomy (Seidlitz et al., 2020). Genome-wide expression queries are possible in Brain Explorer 2 and BrainTrawler. While only brain regions can be used as ROIs for gene expression queries in Brain Explorer 2, BrainTrawler allows the user to define custom ROIs. These custom ROIs can be generated by thresholding the signal of a functional/structural connectivity query, which allows searching for genes in connected regions. Queries for similar gene expression patterns are also available both in BrainTrawler (by signal thresholding) and Brain Explorer 2. Furthermore, BrainTrawler allows genes to be filtered by specific gene ontology (GO) terms. Genome-wide differential queries between regions can currently only be performed using the Allen Brain web page. In addition to a differential search for mouse ISH and human microarray data, differential expression queries can also be executed across mouse ISH and human microarray data, allowing users to compare the fold change for both species in each gene.

#### 4.9.6 Which genes are co-expressed highly with gene X?

Co-expression of specific gene sets can be used to distinguish the major cell classes in the human brain (Oldham et al., 2008). Currently, BrainTrawler and Brain Explorer 2 allow querying for similar expression. In BrainTrawler, this is realized by thresholding an individual expression map and creating an ROI to query, while Brain Explorer 2 simply provides a “Similar Expression” query option.

#### 4.9.7 Does the co-expression network of a set of genes follow structural connectivity?

Gene co-expression and structural connectivity networks can be compared to study neural development and neurodegenerative diseases (Forest et al., 2017; Timonidis et al., 2021). BrainTrawler is the only tool that allows the comparison of multiple networks. However, it does not offer gene co-expression network data and the integration of user-provided co-expression data in a self-hosted BrainTrawler instance is nontrivial (see section on data export and extensibility).

#### 4.9.8 How does functional connectivity change between patients suffering from condition C and healthy controls?

Often, fMRI studies compare functional connectivity between patients and healthy controls to study neuropathology (Hill et al., 2020). Resting-state connectivity for healthy controls can be accessed in SIIBRA, Neurosynth, and BrainTrawler. However, complementary cohorts of diseased patient data are not available in any tool. Neurosynth can be used to access meta-analytic brain activation maps, which also include terms for various conditions, such as schizophrenia. However, these activation maps cannot replace patient–control study data.

#### 4.9.9 Which genes show expression patterns that correlate with a task-specific brain activation?

Neuropsychiatric disorders, such as depression, are known to alter task-evoked brain activation. The discovery of expression patterns that correlate with this activation can guide the development of more targeted therapies (Cai et al., 2024; Komorowski et al., 2022). Unfortunately, no tool considered in this report allows for such comparisons.

#### 4.9.10 Which data are available for region Y?

When studying a brain region, it is important to find datasets that measured data there. The most powerful tool for this task is SIIBRA Explorer. While many data features cannot be visualized directly in the web application, all of them can be accessed through the EBRAINS knowledge graph. As an alternative, BrainTrawler offers a way to visually inspect the region coverage of its transcriptomic database, as shown in Figure 6c. However, connectivity data cannot be explored this way.

### 4.10 Limitations of brain data exploration software

Data exploration software can serve as a means to generate hypotheses based on multi-modal data. By offering general-purpose query and filtering options, exploration software allows researchers to perform data searching on their own with significantly reduced time investment compared with manual programming. Then, researchers can directly export the filtered data from exploration tools and use it as input for subsequent analysis steps. In addition, these tools commonly offer visual data exploration capabilities (e.g., via 3D visualizations or charts) which help users gain understanding about patterns and structure in data.

However, it is important to understand that data exploration is a means for hypothesis generation, not for hypothesis testing. Such testing relies on rigorous statistical analysis, which data exploration platforms do not provide. In addition, all brain data exploration platforms come with certain limitations. First, data pre-processing and computations behind queries are often not communicated clearly. This can be alleviated by developers by providing extensive and thorough documentation. Second, these platforms offer less flexibility in query options compared with custom pipelines. This flexibility is exchanged for the ability to make queries general and applicable to a broad range of questions (see example question in previous sections). Third, the data available within exploration software are often fixed. In principle, this limitation can be overcome by allowing the import and usage of user-procured data. However, we have observed that the import of user data is currently barely feasible across the tools listed in this review. Fourth, brain data exploration software may sacrifice query precision to enhance query speed. Reduced data precision is problematic when analyses directly use exported data from such tools, and needs to be clearly communicated before/during the data export.

All these limitations should be considered when debating whether data exploration software is the correct tool to answer a specific research question.

### 4.11 Analysis tools beyond the scope of this report

The data exploration tools covered in this report aim to give neuroscientists qualitative insights into multi-modal data for hypothesis generation and use graphical user interfaces to simplify access to these data. As a next step, data analysis can provide quantitative answers to research questions generated during exploration using elaborate statistics workflows, as explained by Wei et al. (2022). These workflows can either be set up manually or implemented using dedicated data analysis tools for imaging transcriptomics. Such analysis software is often provided as a software library in popular programming languages such as R, Python, or Matlab, which allows for custom workflows. To complement the detailed review of multi-modal brain data exploration tools, we will briefly mention three useful programmatic brain data analysis tools in this section.

The Imaging Transcriptomics Toolbox (Giacomel et al., 2022; Martins et al., 2021) is an advanced Python library designed to identify gene expression patterns that correlate with specific neuroimaging phenotypes, such as MRI, PET, and EEG, in humans. This toolbox builds upon a suite of established analytical tools in neuroscience, which are listed in the key resources table of the related publication (Giacomel et al., 2022). The abagen toolbox (Markello & Misic, 2021) focuses specifically on standardized preprocessing of data from the AHBA and combining it with arbitrary volumetric or surface parcellations to generate brain-region-by-gene expression matrices. These parcellations can be derived from various neuroimaging data (e.g., functional connectivity; Yeo et al., 2011). Additionally, BrainStat (Larivière et al., 2023) serves as a versatile Python and Matlab toolbox, facilitating statistical analysis and the genetic, meta-analytic, and histological decoding of neuroimaging data. It interfaces with prominent brain atlases, such as the BigBrain Atlas and the Allen Human Brain Atlas, and accommodates various data formats, providing interactive visualization functions.

## 5 Summary and Outlook

In this review, we have summarized data exploration tools for transcriptomic and neuroimaging data. We have evaluated them with regard to the available species, data modalities, data integration approaches, visualizations, and discussed extensions with user data. Furthermore, tools were recommended for different scientific questions based on the available data and features. Lastly, we recommended data analysis tools for subsequent investigations of similar data.

Most investigated tools are specialized for a subset of data exploration tasks. SIIBRA Explorer provides access to numerous parcellations and is linked to a large database, but provides few detailed query options, especially regarding transcriptomics data. Brain Explorer 2 gives users detailed access to Allen Brain Institute expression and axonal projection data for mouse and human, including interactive visualizations, but is limited to four datasets and cannot be extended with user data. With BrainTrawler, users can query transcriptomics and network data with arbitrary ROIs and access expression data on a cell type level. However, only one connectivity dataset per species is available, and activation data cannot be accessed. Neurosynth provides a unique meta-analytical activation database, derived from multiple thousands of studies and linked to cognitive terms, but offers only a single workflow for gene expression data. In LinkRbrain, users can investigate correlations between brain regions, gene expression patterns, and meta-analytical co-activation maps at a glance, but only few workflows are supported.

Although many explorative questions can already be answered using the tools described in this report, there is significant room for improvements that could be exploited in future data exploration tools. When it comes to the type of exploratory questions that can be posed to data, queries linking transcriptomic data to neuroimaging data are still scarce. While some tools provide options for using results of one modality to generate query ROIs for another, there is still significant potential for improvement. New data exploration software could investigate data using entire images of one modality (e.g., the expression of a certain gene) as query inputs and search for spatially correlating signals in another modality (e.g., task fMRI). In addition, future multi-modal queries using connectivity could make stronger use of the network data, for example, by running connectivity-weighted queries on other images or constructing and querying connectivity paths between nodes. Queries could also be improved by going beyond single ROIs and instead querying entire circuits (e.g., the reward circuit) and allowing inspection and comparison of the results on every hierarchical level of these circuits.

Furthermore, we found that the available data often limit the aforementioned queries. In some tools (e.g., BrainTrawler) queries relating spatial gene expression patterns to activation signal are not possible because brain activation data are not available. Future multi-modal brain data exploration tools might focus on filling this gap by integrating data from brain activation data resources such as OpenNeuro (Markiewicz et al., 2021), BrainMap (P. T. Fox & Lancaster, 2002), and NeuroVault (Gorgolewski et al., 2015). This would enable the exploration of correlation between expression patterns and task-specific activation patterns. Second, the integration of diseased patient data, such as functional connectivity in schizophrenics, would open the available workflows to a larger audience. With the commercial availability of high-throughput sequencing platforms and the numerous public data resources, workflows that enable users to easily integrate and explore data would greatly benefit the field of imaging transcriptomics. Third, the increasing resolution and size of publicly available transcriptomics data, such as Siletti et al. (2023) or Yao et al. (2023), hold great opportunities for discovery, but also pose significant challenges on data integration and query performance. This is especially true for spatial transcriptomics, where large-scale tissue profiling with cellular labeling is currently emerging (Schroeder et al., 2025). Future exploration tools could leverage this leap in resolution and integrate such data with other high-resolution data, such as spatial proteomics or epigenomics, electron microscopy or calcium imaging to enable multi-modal hypothesis generation on a micro scale.

Future data exploration software could also be improved in aspects beyond the available queries and data. As an example, only Neurosynth offers a dedicated workflow that can integrate user-provided data with the Neurosynth Decoder. Incorporating user data would immensely improve usefulness, as it gives flexibility to the user. Complementary programmatic interfaces, like the ones offered by SIIBRA and Neurosynth, could be leveraged by future tools to allow easy integration of a tools’ functionality into custom scripts and workflows. Furthermore, such interfaces provide increased reproducibility, as they allow users to explicitly write down how they obtained an exploratory result.

Finally, the multi-modal data integrated in the discussed software could be a useful asset for artificial intelligence, both for training on multi-modal data (e.g., for pattern mining) and for enhancing human-centric workflows with AI in the loop (e.g., by enabling natural language commands) (Bühler et al., 2025). We hope that these findings will prove useful and informative to future developments in brain data exploration software.

## Data and Code Availability

Data and code used for the comparison in Section 4.9 can be found on Zenodo (https://doi.org/10.5281/zenodo.17973995).

## Author Contributions

This manuscript was conceptualized by Tobias Peherstorfer with the help of Bianca Burger, Sophia Ulonska, and Katja Bühler. Tobias Peherstorfer performed the literature search, comparative evaluation, and writing of the review. Bianca Burger, Sophia Ulonska, Wulf Haubensak, and Katja Bühler critically revised the article.

## Ethics Statement

This work adheres to the ethical guidelines for journal publications put forth by the Committee on Publication Ethics (COPE) and the International Committee of Medical Journal Editors (ICMJE). The authors affirm that this work is original and does not contain instances of plagiarism, fabrication, or falsification of data. In the creation of this manuscript, AI tools have not been used to generate content, but have been used to improve language. The authors take full responsibility for the content, including portions revised using AI.

## Funding

The VRVis GmbH is funded by BMIMI, BMWET, Tyrol, Vorarlberg, and Vienna Business Agency in the scope of COMET—Competence Centers for Excellent Technologies (911654) which is managed by FFG. Wulf Haubensak was supported by FWF COE-16B, FWF/DFG DACH I-5074B, and Boehringer Ingelheim.

## Supplementary Material

Supplementary Material
